# The need for area under the curve measurements in the field of ganciclovir therapeutic drug monitoring in children: a case report

**DOI:** 10.1186/s12879-021-06828-8

**Published:** 2021-11-08

**Authors:** Xavier Duval, Florian Lemaitre, Sophie Pertuisel, Jamie Probert, Virginie Gandemer, Marie-Clémence Verdier, Camille Tron

**Affiliations:** 1 Laboratory of Clinical Pharmacology, University Hospital of Rennes, 2 Rue Henri le Guilloux, 35000 Rennes, France; 2 Inserm, EHESP, Irset (Institut de Recherche en Santé, Environnement et Travail), UMR_S 1085, University of Rennes, 35000 Rennes, France; 3 Department of Paediatric Hemato-Oncology, University Hospital of Rennes, 35000 Rennes, France

**Keywords:** Therapeutic drug monitoring, Antiviral therapy, Cytomegalovirus (CMV), Pharmacokinetics, Hematopoietic stem cell transplantation, Pediatrics

## Abstract

**Background:**

Ganciclovir pharmacokinetics is characterized by a high variability in drug exposure. Usually, monitoring of ganciclovir exposure is performed by measuring trough concentration. However, due to the specificity of pediatric pharmacokinetics, trough concentration measurements may not be a relevant surrogate of ganciclovir exposure. Area under the curve of concentration (AUC) may be a more appropriate biomarker.

**Case presentation:**

We report the case of 3.6-year-old boy with Emberger syndrome with a cytomegalovirus reactivation occurring after allogenic hematopoietic stem cell transplantation. After a few days of treatment with intravenous ganciclovir, sub-therapeutic trough ganciclovir concentrations were measured (< 0.5 µg/mL) and viral load still increased. Ganciclovir dosage was increased by two-fold to deal with this treatment failure. Trough concentrations remained sub-therapeutic. The patient had hematologic disorder therefore it was decided to estimate ganciclovir AUC to assess more accurately drug exposure before any further dosage modification. AUC_0–12 h_ was measured at 51 μg h/mL, which was within the therapeutic range (40–60 μg h/mL). Afterward, viral load decreased and became undetectable.

**Conclusions:**

This case report highlights that monitoring ganciclovir exposure based on AUC should be performed to tailor drug dosage in order to improve treatment efficacy and safety in pediatric patients.

## Background

Ganciclovir is the treatment of choice for Cytomegalovirus (CMV) reactivation in immunocompromised patients [1]. Its pharmacokinetics is characterized by a high interpatient variability [2, 3]. Therefore, therapeutic drug monitoring (TDM) of ganciclovir is valuable to individualize dosing regimens [4]. Measuring trough concentration (C_0_) in plasma is the standard approach as a proxy for the antiviral drug exposure. Area under the curve of concentration versus times (AUC) measurements is considered to be the best surrogate for drug exposure. However this parameters is rarely used for ganciclovir TDM in clinical practice while population pharmacokinetic modeling removed practical huddles that used to limit access to AUC-based TDM [5]. Due to the specificity of pediatric pharmacokinetics, trough concentration measurements may not be a relevant exposure biomarker for ganciclovir and AUC evaluation may be more appropriate in this context [2–4].

## Case presentation

To emphasize this statement, we report the case of a 3.6-year-old boy with Emberger syndrome. This syndrome is associated with an increased risk of myelodysplastic syndrome, familial acute myeloid leukemia and immune deficiency. Therapeutic management is limited to prophylactic antimicrobial treatment and pre-emptive allogenic hematopoietic stem cell transplantation (allogenic HSCT).

**Fig. 1:**
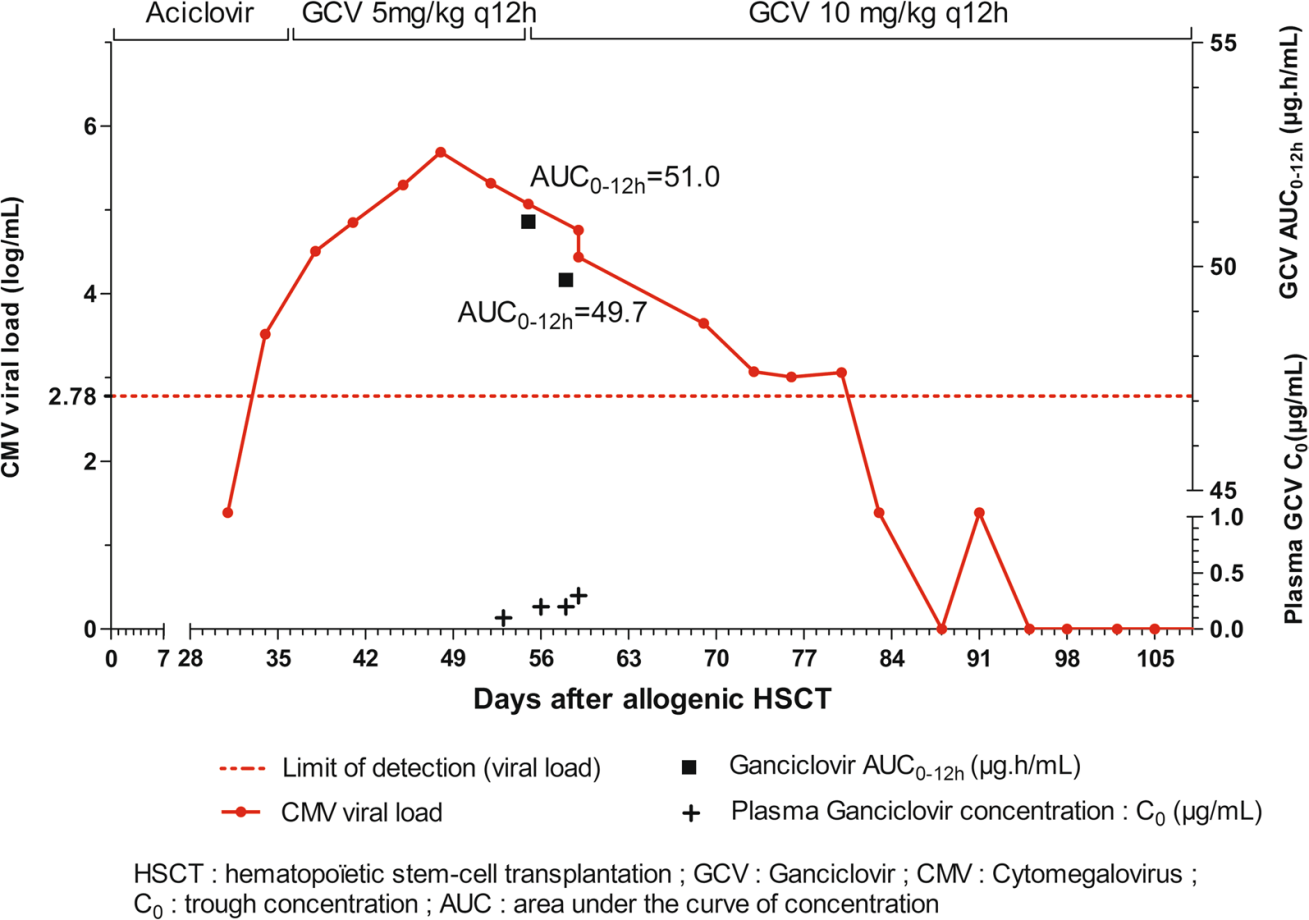
Course of CMV viral load and GCV concentrations. *HSCT* hematopoietic stem-cell transplantation, *GCV* Ganciclovir, *CMV* Cytomegalovirus, *C*_*0*_ trough concentration, *AUC* area under the curve concentration

In June 2020, the patient received an allogenic haploidentical HSCT. After transplantation, he received ciclosporine, mycophenolic acid and post-transplant cyclophosphamide as immunosuppressive therapy. In addition, prednisone was introduced at day-21 post transplantation to treat grade 3 acute graft-versus host disease and 5 days later ruxolitinib was introduced because of progression. Posaconazole was also started at day-26 and cotrimoxazole as a prophylaxis of pneumocystosis at day-48. Thirty-four days after transplantation, a CMV reactivation was diagnosed with a positive CMV viral load at 3.52 log copies/mL. Intravenous ganciclovir was introduced (5 mg/kg q12h; 70 mg q12h) to prevent morbidity and mortality of the infection in this clinical setting. At the same period, pancytopenia has been observed and might have been worsened by several of the drugs received by the patient including ganciclovir. The patient was then administered G-CSF injections every 3 days. After 3 weeks of ganciclovir treatment, the viral load did not decrease (Fig. 1). Low drug exposure and UL97 or UL54 virological resistance were suspected. Trough ganciclovir concentrations were sub-therapeutic (< 0.5 µg/mL) (Fig. 1) and the resistance mutation analysis was negative meaning that treatment failure was likely due to drug underexposure. The patient had a rapid renal clearance (creatinine clearance (CrCl): 220.5 mL/min using Schwartz formula [6]) so it was decided to conduct an AUC-driven ganciclovir dosage adaptation. The dosage was thus increased to 10 mg/kg q12h (140 mg q12h). Exposure was assessed by collecting five plasma concentrations between drug intakes and AUC_0–12 h_ was estimated using a non-compartmental approach based on five sampling times: pre-dose, and then 1, 2, 4 and 6 h post-dose). As displayed in Fig. 1, whereas trough concentrations remained below the usual therapeutic threshold (e.g. < 0.5 µg/mL) [7–9] peak concentrations was high and AUC_0–12 h_ was close to 50 µg h/mL, which was within the therapeutic range (40–60 µg h/mL) suggested by some authors to treat CMV infection [10, 11]. Eventually, viral load decreased quickly after 6 days at this dosage and became undetectable after 52 days. Two months later, viral load has remained undetectable. No significant change in the haematological parameters was noted after ganciclovir dosage modification.

## Discussion and conclusions

This case report emphasizes that TDM of ganciclovir is highly relevant to improve treatment efficacy in the setting of CMV pediatric infection. Standard dosage based on body weight is not a relevant strategy to deal with the high inter-patient variability of ganciclovir pharmacokinetics in pediatrics given the rapid drug clearance in this sub-population [2]. During the 3-weeks period without TDM, ganciclovir exposure was low in this child and CMV replication was not controlled. For practical reasons, TDM is usually based only on the measurement of trough plasma concentration since AUC calculation requires to collect several blood samples. Invasiveness of this approach can be a hurdle to its implementation. However, some teams showed that AUC can be used for TDM in clinical practice complementarily to trough or peak concentrations [5]. In addition, after allogenic HSCT, patients benefit from in-patient monitoring and samples can be collected through a central line. As reported by other teams [4, 9], in this patient, trough concentrations appeared poorly correlated to AUC. Indeed, after dosage adjustment, trough concentrations minimally changed while therapeutic AUC_0–12 h_ was obtained. This is a key point since ganciclovir dosage would have probably been increased beyond 10 mg/kg to reach the target therapeutic range of trough concentrations (> 0.5 to 3 µg/mL) [7–9]. In pediatric patient, dosing ganciclovir according to trough concentrations may then preclude safety of the drug. Although the threshold of AUC associated with toxicity is not fully elucidated [12], the corresponding AUC_0–12 h_ would certainly have been very high (above 60 µg h/mL) resulting in a potential exacerbation of the pancytopenia. AUC measurements allowed dealing with the clinical issue, which was reaching a negative viremia while limiting hematologic disorders coming from ganciclovir overexposure. This case report highlights that monitoring ganciclovir exposure based on AUC should be performed to tailor drug dosage in pediatric patients. It is a useful tool for clinician to increase treatment success and to prevent adverse events.

## Data Availability

Data sharing is not applicable to this article as no datasets were generated or analysed during the current study.

## Abbreviations

CMV: Cytomegalovirus
TDM: Therapeutic drug monitoring
C_0_: Trough concentration
AUC: Area under the curve of concentration versus times
allogenic HSCT: Allogenic hematopoietic stem cell transplantation
CrCl: Creatinine clearance

## References

[1] Vora SB, Englund JA (2015). Cytomegalovirus in immunocompromised children. Curr Opin Infect Dis.

[2] Launay E, Théôret Y, Litalien C, Duval M, Alvarez F, Lapeyraque A-L (2012). Pharmacokinetic profile of valganciclovir in pediatric transplant recipients. Pediatr Infect Dis J.

[3] Vethamuthu J, Feber J, Chretien A, Lampe D, Filler G (2007). Unexpectedly high inter- and intrapatient variability of Ganciclovir levels in children. Pediatr Transplant.

[4] Stockmann C, Roberts JK, Knackstedt ED, Spigarelli MG, Sherwin CM (2015). Clinical pharmacokinetics and pharmacodynamics of ganciclovir and valganciclovir in children with cytomegalovirus infection. Expert Opin Drug Metab Toxicol.

[5] Märtson A-G, Edwina AE, Burgerhof JGM, Berger SP, de Joode A, Damman K (2021). Ganciclovir therapeutic drug monitoring in transplant recipients. J Antimicrob Chemother.

[6] Schwartz GJ, Brion LP, Spitzer A (1987). The use of plasma creatinine concentration for estimating glomerular filtration rate in infants, children, and adolescents. Pediatr Clin N Am.

[7] Zhang D, Lapeyraque A-L, Popon M, Loirat C, Jacqz-Aigrain E (2003). Pharmacokinetics of ganciclovir in pediatric renal transplant recipients. Pediatr Nephrol.

[8] Ritchie BM, Barreto JN, Barreto EF, Crow SA, Dierkhising RA, Jannetto PJ (2019). Relationship of ganciclovir therapeutic drug monitoring with clinical efficacy and patient safety. Antimicrob Agents Chemother.

[9] Giménez E, Solano C, Azanza JR, Amat P, Navarro D (2014). Monitoring of trough plasma ganciclovir levels and peripheral blood cytomegalovirus (CMV)-specific CD8 ^+^ T cells to predict CMV DNAemia Clearance In Preemptively Treated Allogeneic Stem Cell Transplant Recipients. Antimicrob Agents Chemother.

[10] Jorga K, Reigner B, Chavanne C, Alvaro G, Frey N (2019). Pediatric dosing of ganciclovir and valganciclovir: how model-based simulations can prevent underexposure and potential treatment failure. CPT Pharmacomet Syst Pharmacol.

[11] Åsberg A, Bjerre A, Neely M (2014). New algorithm for valganciclovir dosing in pediatric solid organ transplant recipients. Pediatr Transplant.

[12] Franck B, Autmizguine J, Marquet P, Ovetchkine P, Woillard J. Pharmacokinetics, pharmacodynamics and therapeutic drug monitoring of valganciclovir and ganciclovir in transplantation. Clin Pharmacol Ther. 2021;cpt.2431. 10.1002/cpt.2431 34596243

